# Prognostic significance of combined score of fibrinogen and neutrophil-lymphocyte ratio for functional outcome in patients with aneurysmal subarachnoid hemorrhage

**DOI:** 10.3389/fneur.2022.916968

**Published:** 2022-08-16

**Authors:** Yuyang Hou, Hua Li, Hongkuan Yang, Rudong Chen, Jiasheng Yu

**Affiliations:** Department of Neurosurgery, Tongji Hospital, Tongji Medical College, Huazhong University of Science and Technology, Wuhan, China

**Keywords:** inflammation, fibrinogen, neutrophil-lymphocyte ratio, aneurysmal subarachnoid hemorrhage, functional outcome

## Abstract

**Objective:**

To explore the relationship between fibrinogen and neutrophil to lymphocyte ratio (F-NLR) score and functional outcomes after aneurysmal subarachnoid hemorrhage (aSAH).

**Method:**

A retrospective study was conducted that involved all consecutive patients with aSAH admitted to our institution from March 2018 to October 2021. Factors, such as demographics, comorbidities, clinical characteristics, neuroradiological data, and laboratory parameters, were collected from institutional databases. All patients achieved neurological assessment using the modified Rankin Scale (mRS) score 3 months after discharge to clarify the functional outcomes. The results were classified as favorable (mRS score 0–2) and unfavorable (mRS score 3–6). Univariate and multivariable analyses were performed to identify the relevant factors between inflammatory markers and functional outcomes after aSAH. Subsequently, a receiver operating characteristic (ROC) curve analysis was conducted to evaluate the predicting performance of variables. A propensity score match (PSM) was performed to correct imbalances in patients' baseline characteristics.

**Results:**

Finally, 256 patients with aSAH were included in the study cohort. A total of 94 (36.7%) patients had an unfavorable outcome. F-NLR scores were 0 [interquartile range (IQR) 0–1] and 1 (IQR 1–2) in patients with favorable and unfavorable outcomes, respectively (*p* < 0.001). After adjustment, the F-NLR score on admission remained significantly associated with unfavorable outcomes in patients with aSAH. In the multivariable analysis, the F-NLR score was regarded as an independent risk factor of unfavorable outcomes [odds ratio (OR) 3.113, 95% CI 1.755–5.523, *p* < 0.001]. In ROC analysis, the optimal cutoff value of the F-NLR score was 0.5 points. Two cohorts (n = 86 in each group) obtained from PSM with low F-NLR scores (0 points) and high F-NLR scores (1–2 points) were used for analysis. A significantly higher unfavorable functional outcome rate was observed in patients with high F-NLR scores (33.7 vs. 9.3%, *p* < 0.001). The area under the curve (AUC) values of F-NLR scores before and after PSM were 0.767 and 0.712, respectively.

**Conclusion:**

Fibrinogen and neutrophil to lymphocyte ratio score was an independent risk parameter associated with unfavorable functional outcomes at 3 months after aSAH. A higher F-NLR score predicts the occurrence of poor functional outcomes.

## Introduction

Subarachnoid hemorrhage (SAH) is a fatal type of hemorrhagic stroke with high mortality and morbidity, aneurysmal subarachnoid hemorrhage (aSAH) is the cause of 85% of cases of spontaneous SAH (1). Meanwhile, survivors may suffer from different functional deficits that may affect the quality of life and make it difficult to return to work (2, 3). Therefore, investigating prognostic factors for functional outcomes after aSAH is an aspirational destination. Although the specific pathological mechanism of brain injury caused by aSAH remains uncertain. There is accumulating evidence that the inflammatory response may play a crucial role in the pathogenesis of brain injury due to cerebrovascular disease (4, 5). Previous studies have identified several inflammatory markers, such as neutrophil count and platelet-to-lymphocyte ratio, as predictors of functional outcomes after aSAH (6–8). Already established clinical grading scales, such as the World Federation of Neurosurgical Societies (WFNS) and Hunt-Hess scales, were commonly used by neurosurgeons to evaluate the prognosis of patients after aSAH. A combination of plasma fibrinogen and neutrophil to lymphocyte ratio (F-NLR) score that consists of peripheral blood F-NLR is emerging as a novel inflammatory marker. This combined index was widely used in oncology studies (9, 10), but the assessment of the F-NLR score for cerebrovascular disease was not well identified, especially for aSAH. These laboratory parameters are readily available, inexpensive, convenient to apply, and reflect both proinflammatory and procoagulant status. In this retrospective study, we designed to investigate the relationship between F-NLR scores on admission and neurological outcomes of patients at 3 months after aSAH and to assess the predictive value.

## Materials and methods

### Study population

The current study was a single-center retrospective review. From March 2018 to October 2021, all consecutive patients with aASH who were admitted to the Department of Neurosurgery, Tongji Hospital, Tongji Medical College, Huazhong University of Science and Technology were recruited. The inclusion criteria were as follows: (1) spontaneous SAH was identified by using computerized tomography (CT) or lumbar puncture; (2) patients with a definite diagnosis of intracranial aneurysm (IA) by computerized tomography angiography (CTA), digital subtraction angiography (DSA), or surgery; (3) patients who underwent surgical clipping or endovascular treatment for the aneurysm resulting in SAH within 72 h; (4) age ranged from 18 to 80 years old; and (5) adequate laboratory data before treatment could be gathered from medical records. In addition, the exclusion criteria were as follows: (1) patients combined with brain injury or other cerebrovascular disease and the relationship between SAH and IA could not be clarified; (2) patients without treatment for ruptured aneurysm within 72 h; (3) patients who followed up <3 months; (4) underlying cancer, severe kidney, liver dysfunction, or history of autoimmune; (5) inadequate laboratory data were available.

### Choice of treatment

The therapeutic choice was based on the following principles: (1) surgical clipping was preferred for ruptured aneurysms (especially anterior circulation aneurysms) with obvious intracranial hematoma (bleeding >50 ml). (2) Aneurysms with branch vessels emanating from the aneurysm sac and aneurysms located in the middle cerebral artery bifurcation surgical clipping were favored. (3) For patients with low-grade aSAH, both coiling and clipping could be performed, but coiling may be the first consideration. (4) For ruptured aneurysms located in the posterior circulation, embolization is the preferred treatment. In summary, the specific treatment should be discussed in a multidisciplinary manner and based on a comprehensive assessment of individual patient factors, such as age, general health, aneurysm site, aneurysm morphology, and family wishes.

### Laboratory parameters

All laboratory data were determined by peripheral venous blood sampling on admission. Laboratory parameters contained detailed peripheral blood counts of red blood cells, leukocytes, neutrophils, lymphocytes, monocytes, platelet counts, prothrombin time, activated partial thromboplastin time, prothrombin time, fibrinogen, and non-fasting serum glucose. NLR was defined as neutrophil count to lymphocyte count. Fibrinogen and NLR were classified into high and low groups based on the optimal cutoff values according to the receiver operating characteristic (ROC) curves. F-NLR score was defined as follows: (1) score 0, low fibrinogen and low NLR; (2) score 1, low fibrinogen and high NLR or high fibrinogen and low NLR; and (3) score 2, high fibrinogen and high NLR (11). Patients were not treated with steroids and received 2.0 g of tranexamic acid intravenously every 12 h after blood sample collection. Blood samples were drawn within 6 h of admission.

### Clinical characteristics and outcomes

Demographic data of age, gender, and comorbidities, such as hypertension, diabetes mellitus, dyslipidemia, smoking, and drinking, were obtained. Qualified neurosurgeons assessed all patients for initial neurologic status, such as Glasgow Coma Score (GCS), Hunt-Hess grade, and the World Federation of Neurosurgical Societies (WFNS) grade. Based on CT imaging, neuroradiologists observed for the presence of intracerebral hemorrhage (ICH), intraventricular hemorrhage (IVH), or acute hydrocephalus, and a modified Fisher (mFisher) scale was used to assess the extent of SAH to determine the radiologic characteristics. The information on the location of aneurysm was acquired from CTA, DSA, or surgery.

Neurological function outcome was assessed by a qualified neurosurgeon by using the modified Rankin Scale (mRS) score through a semi-structured telephone interview. Depending on the results, a favorable outcome was defined as an mRS score of 0–2, representing a functional independent status, and an unfavorable outcome as an mRS score of 3–6.

Non-ventilator hospital-acquired pneumonia occurs after 48 h of hospital stay and hospital-acquired pneumonia occurs after 48 h of mechanical ventilation. The criteria for defining hospital-acquired pneumonia were as follows.

(1) Radiological signs: Two successive chest radiographs showing new or progressive lung infiltrates, in the absence of a medical history of underlying heart or lung disease, a single chest radiograph is enough.

(2) In addition, at least one of the following signs: body temperature >38.3°C without any other cause and leukocyte counts <4 × 10^9^/L or ≥12 × 10^9^/L.

(3) In addition, at least two of the following signs: purulent sputum, cough, dyspnea, declining oxygenation, increased oxygen requirement, or need for respiratory assistance.

### Statistical analysis

Continuous variables were expressed as mean ± standard deviation (SD) or median (interquartile range, IQR), as appropriate. According to the normality of the distribution of continuous variables, the independent samples t-test or the Mann-Whitney U-test was performed. For categorical variables, frequencies were described and analyzed by the chi-square test or the Fisher exact test, as appropriate.

We selected factors as confounders follow the following principles: (1) a factor had a change in the effect estimate of more than 10%; and (2) a factor was significantly associated with the outcomes of interest. In order to mitigate the interference of potential confounding factors with the results, univariable and multivariable logistic regression analyses were conducted to further illustrate the relationship between functional outcomes 3 months after aSAH and F-NLR scores. In the crude model, no factors were adjusted. In model 1, age and gender were adjusted. In addition to age and gender, model 2 was adjusted for comorbidities, such as hypertension, hyperlipidemia, diabetes mellitus, smoking, and drinking. On the basis of model 2, we further adjusted for variables that might be related to the severity of aSAH, such as Hunt-Hess grade, WFNS grade, mFisher grade, hydrocephalus, ICH, and IVH, in model 3.

All factors with *p* < 0.05 in univariable analysis were included in a backward stepwise multivariable logistic regression to identify independent prognostic factors associated with unfavorable outcomes 3 months after aSAH. In order to understand the performance of prognostic factors better, an ROC curve analysis was performed. The area under the curve (AUC) was calculated to evaluate the performance of each variable. The optimal cutoff value was determined by using the maximization of Youden's index. According to the optimal cutoff value of the F-NLR score, patients were divided into two groups, the low F-NLR score (0 points) group and the high F-NLR score (1–2 points) group. To minimize confounding bias, 1:1 propensity score matching (PSM) was performed with caliper 0.15 to adjust for imbalances of different clinically relevant parameters, such as the presence of ICH and IVH, diabetes mellitus, hydrocephalus, pneumonia, Hunt-Hess grade, WFNS grade, GCS score, and mFisher grade, between the two groups. Statistical analyses were performed using software SPSS 25.0 (SPSS Inc., Chicago, IL, USA). *p* < 0.05 was considered statistically significant.

## Results

### Patient characteristics

**Figure 1:**
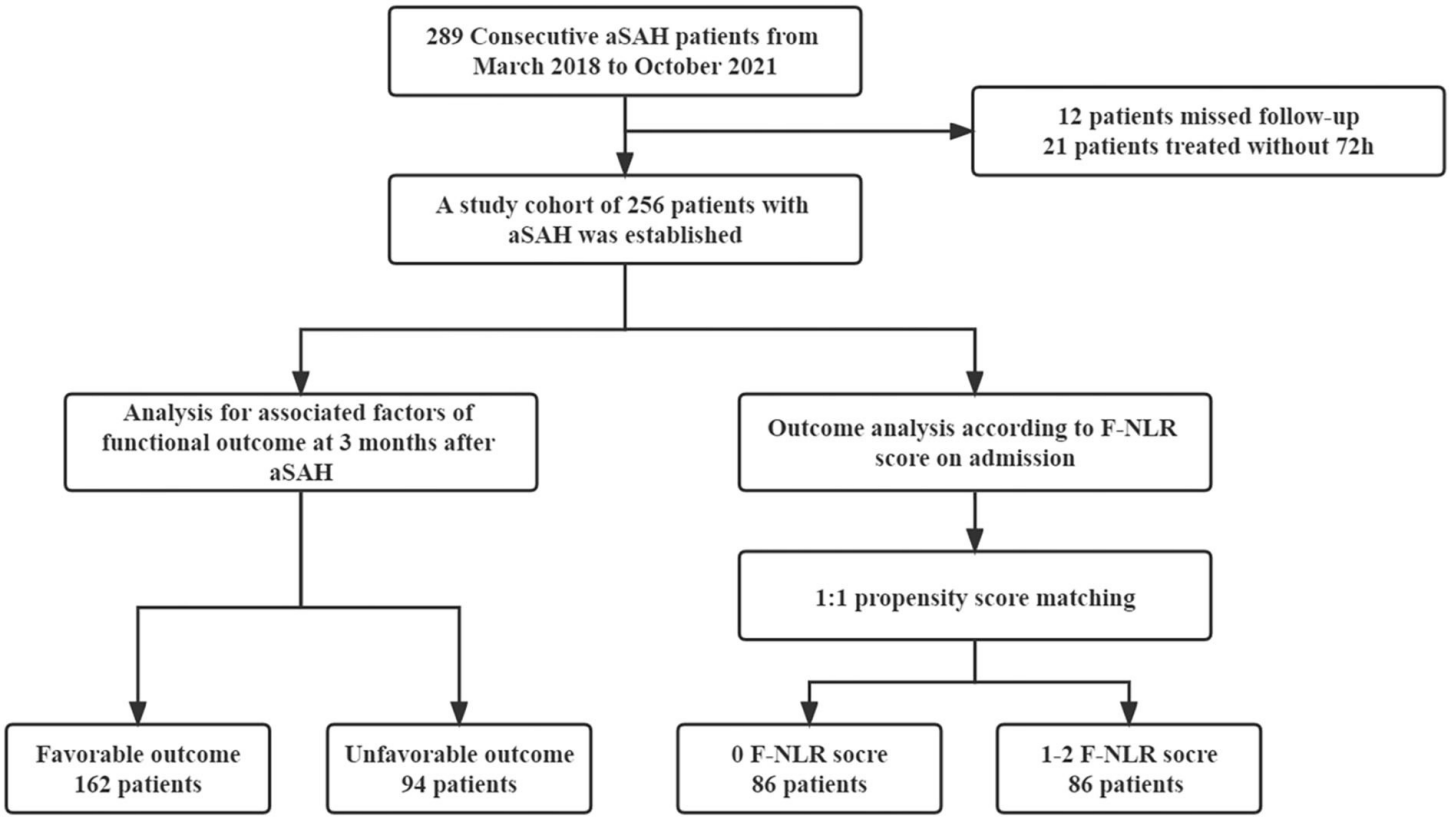
Flowchart of study. aSAH, aneurismal subarachnoid hemorrhage; F, fibrinogen; NLR, neutrophil to lymphocyte ratio.

The study flowchart is shown in Figure 1. Overall, 256 patients with aSAH were enrolled in the final cohort, consisting of 89 (34.9%) men and 167 (65.2%) women. The average age was 56.3 ± 9.0 years. Moreover, 119 (46.5%) patients were combined with hypertension, 53 (20.7%) patients with hyperlipidemia, and 25 (9.8%) patients with diabetes mellitus. In total, 62 (24.2%) patients were current smokers and 66 (25.8%) patients drank alcohol. The median admission H-H grade was 2 (IQR 1–4), the median admission WFNS grade was 1 (IQR 1–3), and the median admission GCS score was 15 (IQR 13–15). The optimal cutoff values of fibrinogen and NLR were 3.7 and 8.1 g/L, respectively. Based on these results, 55 (21.5%) patients were defined as high fibrinogen and 142 (55.5%) patients were defined as having high NLR. In total, 80 (31.2%) patients were treated with endovascular coiling and 176 (68.8%) patients received surgical clipping as treatment. In total, 60 (23.4%) patients were presented with ICH, 80 (31.1%) patients presented with IVH, and the median mFisher grade was 1 (IQR 1–3). Meanwhile, an unfavorable functional outcome was observed in 94 (36.7%) patients and 162 (63.3%) patients obtained favorable functional outcomes. The baseline characteristics are detailed in Table 1.

**Table 1:** Comparison of aSAH patients according to 3-month functional outcome.

**Variables**	**Total (n = 256)**	**Favorable (n = 162)**	**Unfavorable (n = 94)**	***P*-value**
**Demographics**				
Age, Mean ± SD	56.3 ± 9.0	55.4 ± 9.1	57.8 ± 8.8	0.037
Female, n (%)	167 (65.2)	107 (66)	60 (63.8)	0.823
**Comorbidities**				
Hypertension, n (%)	119 (46.5)	71 (43.8)	48 (51.1)	0.299
Hyperlipidemia, n (%)	53 (20.7)	34 (21)	19 (20.2)	1
Diabetes mellitus, n (%)	25 (9.8)	9 (5.6)	16 (17)	0.006
Smoking, n (%)	62 (24.2)	39 (24.1)	23 (24.5)	1
Drinking, n (%)	66 (25.8)	26 (27.7)	40 (24.7)	0.657
**Clinical data**				
Admission Hunt-Hess grade, median (IQR)	2 (1–3)	1 (1–2)	3 (3–4)	<0.001
Admission WFNS grade, median (IQR)	1 (1–3)	1 (1–1)	4 (2–5)	<0.001
Admission GCS score, median (IQR)	15 (13–15)	15 (15–15)	12 (6–14)	<0.001
Hydrocephalus, n (%)	35 (13.7)	9 (5.6)	26 (27.7)	<0.001
Pneumonia, n (%)	65 (25.4)	12 (7.4)	53 (56.4)	<0.001
**Neuroradiological data**				<0.001
Presence of ICH, n (%)	60 (23.4)	17 (10.5)	43 (45.7)	<0.001
Presence of IVH, n (%)	80 (31.2)	26 (16)	54 (57.4)	<0.001
mFisher grade median (IQR)	1 (1–3)	1 (1–2)	3 (2–4)	<0.001
**Aneurysm locations**				0.352
Middle cerebral artery, n (%)	61 (23.8)	40 (24.7)	21 (22.3)	
Anterior cerebral artery, n (%)	14 (5.5)	7 (4.3)	7 (7.4)	
Anterior communicating artery, n (%)	66 (25.8)	40 (24.7)	26 (27.7)	
Posterior communicating artery, n (%)	80 (31.1)	55 (34.0)	25 (26.6)	
Internal carotid artery, n(%)	16 (6.3)	6 (3.7)	10 (10.6)	
Posterior circulation, n (%)	14 (5.5)	11 (6.8)	3 (3.2)	
Others, n (%)	5 (2)	3 (1.9)	2 (2.1)	
**Treatment**				1
Coiling, n (%)	80 (31.2)	51 (31.5)	29 (30.9)	
Clipping, n (%)	176 (68.8)	111 (68.5)	65 (69.1)	
**Laboratory parameters on admission, median (IQR)**				
Glucose, mmol/L	7.1 (6.1–8.6)	6.6 (5.7–7.4)	8.5 (7.3–10.5)	<0.001
Platelet count, ×10^9^/L	201.0 (160.0–253.2)	207.0 (171.0–255.8)	185.0 (151.5–251.0)	0.097
Hemoglobin, g/L	129.0 (117.0–141.0)	129.5 (119.2–139.0)	128.0 (115.2–141.0)	0.368
Red blood cells count, ×10^12^/L	4.3 (3.9–4.6)	4.3 (4.0–4.6)	4.3 (3.8–4.7)	0.900
Leukocytes count, ×10^9^/L	10.8 (8.0–13.7)	9.5 (7.5–11.7)	13.3 (10.7–17.7)	<0.001
Neutrophil count, ×10^9^/L	9.0 (6.4–11.8)	7.7 (5.7–10.0)	11.7 (9.3–15.9)	<0.001
Lymphocyte count, ×10^9^/L	1.0 (0.7–1.3)	1.1 (0.8–1.4)	0.8 (0.6–1.1)	<0.001
Monocyte count, ×10^9^/L	0.5 (0.4–0.7)	0.5 (0.4–0.7)	0.6 (0.4–0.8)	<0.001
Prothrombin time, sec	16.2 (15.5–16.8)	16.2 (15.6–16.8)	16.0 (15.3–16.8)	0.200
Activated partial thromboplastin time, sec	33.5 (31.5–35.9)	33.5 (31.7–36.0)	33.3 (31.3–35.8)	0.547
Fibrinogen, g/L	3.0 (2.7–3.7)	3.0 (2.6–3.5)	3.2 (2.8–3.9)	0.004
NLR	9.7 (5.6–15.9)	7.2 (4.5–11.1)	15.7 (10.4–22.9)	<0.001
**F-NLR score**				<0.001
0	95 (37.1)	87 (53.7)	8 (8.5)	
1	125 (48.8)	66 (40.7)	59 (62.8)	
2	36 (14.1)	9 (5.6)	27 (28.7)	

*aSAH, aneurismal subarachnoid hemorrhage; IQR, interquartile range; WFNS, World Federation of Neurosurgical Societies; GCS, Glasgow Coma Scale; ICH, intracerebral hemorrhage; IVH, intraventricular hemorrhage; mFisher, modified Fisher; NLR, neutrophil to lymphocyte ratio; F, fibrinogen*.

### Predictive factors for functional outcomes in patients with aSAH

To determine the prognostic factors that were independently associated with unfavorable functional outcomes 3 months after aSAH, univariable and multivariable logistic regression models were performed. In the crude model, no parameters were adjusted, and blood biomarkers were related to unfavorable outcomes after aSAH. After adjusting for potential confounding factors, such as age, sex, comorbidities (hypertension, hyperlipidemia, diabetes mellitus, smoking, and drinking), and disease severity indicators (Hunt-Hess grade, WFNS grade, mFisher grade, hydrocephalus, ICH, and IVH), neutrophil count, lymphocyte count, fibrinogen, NLR, and F-NLR score continued to be associated with unfavorable outcomes in patients with aSAH (Table 2). Considering the multicollinearity problem, we performed a linearity check, the variance inflation factors of the WFNS score and GCS were 13.57 and 10.19, respectively, and the variance inflation factors of all remaining variables were <5. All variables that demonstrated statistical significance (*p* < 0.05) in previous univariable analysis were included in multivariable logistic regression, and then we performed regression analysis excluding WFNS score and GCS, respectively. The results are shown in Table 3. It could be noticed that the results of these three analyses did not differ substantially. As shown in Table 3, F-NLR score [odds ratio (OR) 7.851, 95% CI 3.400–18.129, *p* < 0.001], Hunt-Hess grade (OR 3.118, 95% CI 1.861–5.223, *p* < 0.001), mFisher grade (OR 2.115, 95% CI 1.231–3.634, *p* = 0.007), blood glucose (OR 1.409, 95% CI 1.097–1.810, *p* = 0.007), and hospital-acquired pneumonia (OR 7.691, 95% CI 2.349–25.181, *p* = 0.001) are independent prognostic factors for unfavorable outcomes after aSAH.

**Table 2:** Association of laboratory parameters with functional outcome at 3 months after aSAH.

**Characteristic**	**Crude**		**Model 1**		**Model 2**		**Model 3**	
	**OR (95% CI)**	***P*-value**	**OR (95% CI)**	***P*-value**	**OR (95% CI)**	***P*-value**	**OR (95% CI)**	***P*-value**
Neutrophil count	1.353 (1.242–1.437)	<0.001	1.376 (1.260–1.504)	<0.001	1.377 (1.259–1.507)	<0.001	1.179 (1.050–1.323)	0.005
Lymphocyte count	0.257 (0.134–0.491)	<0.001	0.259 (0.134–0.499)	<0.001	0.224 (0.113–0.444)	<0.001	0.299 (0.112–0.800)	0.016
Fibrinogen	1.559 (1.116–2.178)	0.009	1.516 (1.087–2.116)	0.014	1.473 (1.062–2.045)	0.020	1.726 (1.020–2.919)	0.042
NLR	1.172 (1.119–1.228)	<0.001	1.175 (1.120–1.233)	<0.001	1.179 (1.124–1.236)	<0.001	1.106 (1.036–1.180)	0.002
F-NLR score	6.058 (3.608–10.172)	<0.001	6.071 (3.604–10.226)	<0.001	5.443 (2.513–11.787)	<0.001	10.574 (3.147–35.526)	<0.001

*Model 1: Adjusted for age, gender*.

*Model 2: Model 1 plus comorbidities (hypertension, hyperlipidemia, diabetes mellitus, smoking and drinking)*.

*Model 3: Model 2 plus variables related to disease severity (Hunt-Hess grade, WFNS grade, mFisher grade, hydrocephalus, ICH and IVH)*.

*aSAH, aneurismal subarachnoid hemorrhage; OR, odds ratio; CI, confidence interval; WFNS, World Federation of Neurosurgical Societies; mFisher, modified Fisher; ICH, intracerebral hemorrhage; IVH, intraventricular hemorrhage; NLR, neutrophil to lymphocyte ratio; F, fibrinogen*.

**Table 3:** Multivariable analysis of parameters associated with unfavorable outcome after aSAH.

**Characteristic**	**All parameters**		**Excluding WFNS score**		**Excluding GCS**	
	**OR (95% CI)**	***P*-value**	**OR (95% CI)**	***P*-value**	**OR (95% CI)**	***P*-value**
Age	0.975 (0.922–1.031)	0.372	0.974 (0.923–1.029)	0.352	0.974 (0.923–1.029)	0.352
Diabetes mellitus	2.240 (0.363–13.829)	0.385	2.259 (0.381–13.408)	0.370	2.259 (0.381–13.408)	0.370
IVH	2.529 (0.904–7.073)	0.077	2.529 (0.904–7.073)	0.077	2.529 (0.904–7.073)	0.077
ICH	2.220 (0.690–7.144)	0.181	2.215 (0.700–7.088)	0.176	2.215 (0.700–7.088)	0.176
Hydrocephalus	0.439 (0.090–2.138)	0.308	0.379 (0.080–1.808)	0.224	0.379 (0.080–1.808)	0.224
GCS	1.356 (0.859–2.140)	0.191	1.055 (0.777–1.433)	0.730		
WFNS	1.320 (0.715–2.436)	0.375			1.161 (0.605–2.228)	0.653
Leukocytes count	1.025 (0.894–1.175)	0.722	1.012 (0.898–1.141)	0.845	1.012 (0.898–1.141)	0.845
Neutrophil count	0.937 (0.364–2.411)	0.892	0.931 (0.270–3.216)	0.910	0.931 (0.270–3.216)	0.910
Monocyte count	1.059 (0.034–32.867)	0.974	1.108 (0.061–20.241)	0.945	1.108 (0.061–20.241)	0.945
Lymphocyte count	1.155 (0.174–7.655)	0.881	0.995 (0.087–11.422)	0.997	0.995 (0.087–11.422)	0.997
Fibrinogen	1.167 (0.720–1.892)	0.532	1.202 (0.761–1.899)	0.429	1.202 (0.761–1.899)	0.429
NLR	1.013 (0.908–1.129)	0.819	1.015 (0.944–1.093)	0.683	1.015 (0.944–1.093)	0.683
Hunt-Hess grade	3.118 (1.861–5.223)	<0.001	3.118 (1.861–5.223)	<0.001	3.118 (1.861–5.223)	<0.001
mFisher grade	2.115 (1.231–3.634)	0.007	2.115 (1.231–3.634)	0.007	2.115 (1.231–3.634)	0.007
Glucose	1.409 (1.097–1.810)	0.007	1.409 (1.097–1.810)	0.007	1.409 (1.097–1.810)	0.007
Pneumonia	7.691 (2.349–25.181)	0.001	7.691 (2.349–25.181)	0.001	7.691 (2.349–25.181)	0.001
F-NLR score	7.851 (3.400–18.129)	<0.001	7.851 (3.400–18.129)	<0.001	7.851 (3.400–18.129)	<0.001

*aSAH, aneurismal subarachnoid hemorrhage; OR, odds ratio; CI, confidence interval; GCS, Glasgow Coma Score; WFNS, World Federation of Neurosurgical Societies; mFisher, modified Fisher; ICH, intracerebral hemorrhage; IVH, intraventricular hemorrhage; NLR, neutrophil to lymphocyte ratio; F, fibrinogen*.

### Association of F-NLR score with functional outcomes

**Figure 2:**
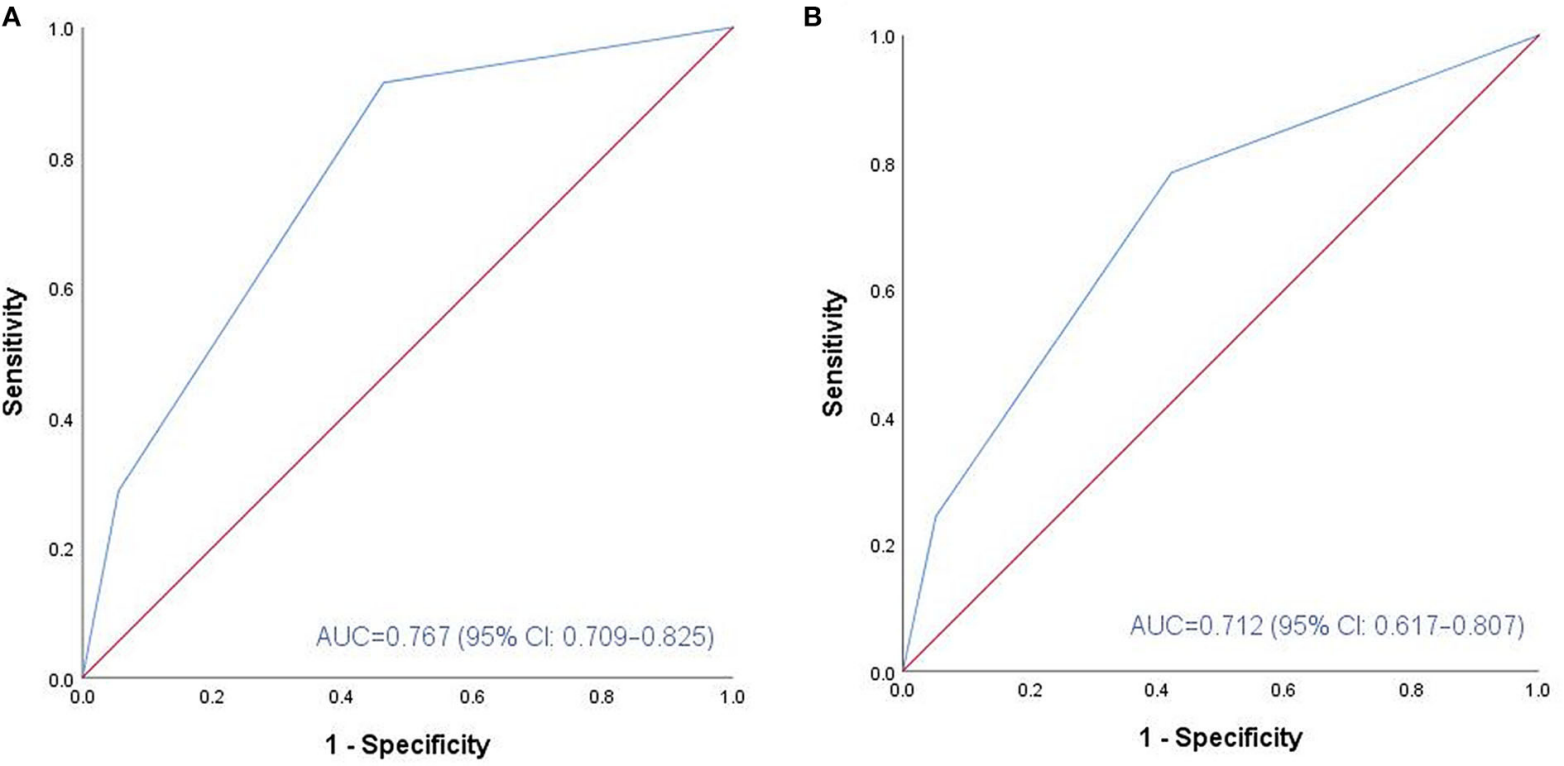
ROC analysis of the association between F-NLR score and functional outcome at 3 months before and after PSM. **(A)** Before PSM, F-NLR score showed promising predictive performance (AUC: 0.767, 95% CI: 0.709-0.825; P < 0.001). **(B)** After PSM, F-NLR score still performed well (AUC: 0.712, 95% CI: 0.617-0.807; P < 0.001). ROC, receiver operating characteristic; aSAH, aneurismal subarachnoid hemorrhage; F, fibrinogen; NLR, neutrophil to lymphocyte ratio; PSM, propensity score matching; AUC, area under the curve.

In ROC analysis, the F-NLR score of 0.5 points was assessed as the optimal cutoff value. Based on this result, patients were divided into two groups, the low F-NLR score (0 points) group and the high F-NLR score (1–2 points) group. Before PSM, patients with high F-NLR scores had worse clinical status on admission. Considering the confounding factors between the two groups, PSM was conducted and two well-balanced cohorts (n = 86 in each group) were available for analysis of functional outcomes after aSAH was obtained. As Table 4 lists, no significant differences are observed in the clinical factors between the two groups after PSM. Compared with patients with low F-NLR scores, a significantly higher proportion of patients with high F-NLR scores had unfavorable functional outcomes 3 months after aSAH (33.7 vs. 9.3%, *p* < 0.001). The AUC values of F-NLR scores were 0.767 (95% CI: 0.709–0.825) and 0.712 (95% CI: 0.617–0.807) before and after PSM, respectively (Figure 2). The results confirmed that the F-NLR scores showed promising prognostic performance for functional outcomes at 3 months in patients with aSAH.

**Table 4:** The clinical characteristics and outcomes in aSAH patients before and after PSM.

**Characteristic**	**Before PSM**				**After PSM**			
	**All patients**	**Low F-NLR score**	**High F-NLR score**	***P*-value**	**All patients**	**Low F-NLR score**	**High F-NLR score**	***P*-value**
**N**	256	95	161		172	86	86	
Diabetes mellitus, n (%)	25 (9.8)	7 (7.4)	18 (11.2)	0.321	15 (8.7)	7 (8.1)	8 (9.3)	1
Admission Hunt-Hess grade, median (IQR)	2 (1–3)	1 (1–2)	3 (1–3)	<0.001	1 (1–2)	1 (1–2)	1 (1–2)	0.144
Admission WFNS grade, median (IQR)	1 (1–3)	1 (1–1)	2 (1–4)	<0.001	1 (1–1.75)	1 (1–1)	1 (1–2)	0.326
Admission GCS score, median (IQR)	15 (13–15)	15 (15–15)	14 (10–15)	<0.001	15 (14–15)	15 (15–15)	15 (14–15)	0.212
Hydrocephalus, n (%)	35 (13.7)	7 (7.4)	28 (17.4)	0.024	16 (9.3)	7 (8.1)	14 (16.3)	0.794
Pneumonia, n (%)	65 (25.4)	14 (14.7)	51 (31.7)	0.003	26 (15.1)	12 (14.0)	13 (18.1)	0.832
Presence of ICH, n (%)	60 (23.4)	8 (8.4)	52 (32.3)	<0.001	20 (11.6)	8 (9.3)	12 (14.0)	0.476
Presence of IVH, n (%)	80 (31.2)	16 (16.8)	64 (39.8)	<0.001	39 (22.7)	16 (18.6)	23 (26.7)	0.274
mFisher grade median (IQR)	1 (1–3)	1 (1–2)	2 (1–3)	<0.001	1 (1–2)	1 (1–2)	1 (1–2)	0.251
**Functional outcome**				<0.001				<0.001
Favorable	162 (63.3)	87 (91.6)	75 (46.6)		135 (78.5)	78 (90.7)	57 (66.3)	
Unfavorable	94 (36.7)	8 (8.4)	86 (53.4)		37 (21.5)	8 (9.3)	29 (33.7)	

*aSAH, aneurismal subarachnoid hemorrhage; PSM, propensity score matching; F, fibrinogen; NLR, neutrophil to lymphocyte ratio; IQR, interquartile range; WFNS, World Federation of Neurosurgical Societies; GCS, Glasgow Coma Scale; ICH, intracerebral hemorrhage; IVH, intraventricular hemorrhage; mFisher, modified Fisher*.

## Discussion

Neuroinflammation has emerged as a new conversation in neurological diseases, especially cerebrovascular diseases (12, 13). Inflammatory markers collected from peripheral blood are easily accessible, almost non-invasive and inexpensive, and could be applied conveniently. These parameters have attracted the attention of clinicians to predict the prognosis of aSAH (14–16). The F-NLR score is a novel marker of inflammation composed of peripheral blood fibrinogen content and NLR. This combined index was extensively used in oncology research (17, 18). To the best of our knowledge, research on the prognostic significance of the F-NLR score in aSAH is limited. Yang et al. (11) had revealed the usefulness of F-NLR scores in ICH, it was an independent risk factor for 3-month functional outcomes. Despite the difference in disease entity, similar result was observed in our study. In multivariable logistic regression analysis, the F-NLR score on admission was identified as an independent risk factor for unfavorable functional outcomes at 3 months in patients with aSAH, and its prognostic predictive ability was demonstrated by ROC analysis. Minimizing confounding bias by using PSM, aSAH patients with higher F-NLR scores are more likely to experience unfavorable functional outcomes.

Fibrinogen is an acute-phase protein synthesized by hepatocytes in the liver. It is a multipotent protein with essential effects in coagulation, inflammation, and tissue repair (19). A key change in the extracellular microenvironment in neuroinflammation is the substantial extravasation of the fibrinogen into the central nervous system (CNS) through the blood-brain barrier (BBB) with increased permeability (20). Although fibrinogen is not normally found in the healthy CNS, it is deposited in a variety of neurological disorders. Fibrinogen is engaged in the blood coagulation process and triggers neuroinflammation by activating microglia and facilitating the recruitment and activation of peripheral inflammatory macrophages to the CNS for transformation into fibronectin (21). Hemostasis and fibrinolysis after SAH might have an impact on the content of fibrinogen (22). Xie et al. (23) demonstrated that the reduction of admission serum fibrinogen levels could improve the prognosis of aSAH patients with high Hunt-Hess grade.

A primary factor affecting functional outcomes after SAH is the early brain damage (EBI) caused during the initial hemorrhage (24). Previous studies have demonstrated that coagulation disorders emerge early after SAH and may lead to EBI (25). Within 72 h after aneurysm rupture, markers of platelet activation and inflammation persisted significantly increased in patients with low Hunt-Hess grade and may be involved in EBI (26). Xie et al. (23) discovered that patients with SAH had increased fibrinogen levels on admission as compared to the normal healthy population. Increased fibrinogen levels on admission might be the result of fibrinolysis after SAH. Fibrinogen may be involved in the pathogenesis of EBI after aSAH thereby leading to poor functional outcomes. Hypofibrinogenemia may be caused by excessive fibrinogen consumption due to complications following SAH, such as sepsis or diffuse intravascular coagulation, and it was not observed in this study.

Although several previous studies had identified NLR as a biomarker for worse functional outcomes after aSAH (7, 8, 15), the specific relationship among them remained obscure and may be due to the following reasons. Unfavorable outcomes may be attributed to complications, such as delayed cerebral ischemia (DCI) or cerebral vasospasm (CVS). Bleeding in the subarachnoid space triggers a systemic cellular response that elicits leukocytosis and neutrophilia, resulting in brain damage and DCI (27, 28). Leukocytosis as an independent predictor of DCI in patients with SAH has been reported in a former study (29). Moreover, several inflammatory molecules and signaling pathways have been implicated in the progression of CVS in patients with SAH (30). F-NLR score is a composite index reflecting both the proinflammatory and procoagulant status that may provide additional information to clinical scales, which is already established, such as the Hunt-Hess and WFNS grading scales, in an attempt to improve the predictive accuracy of prognosis for aSAH. Larger sample size multicenter prospective randomized trials will be needed in the future to investigate the role of F-NLR in aSAH.

### Limitations

There are several limitations to our study that merit consideration. Not all patients are admitted to our institution within a similar time after rupture of an aneurysm, which might cause unknown bias in laboratory parameters. However, it was inevitable, each patient resides in a very different area, and the length of time is necessary to visit the clinic varies. Frequent testing of these laboratory parameters and calculating averages may be able to reduce bias due to different time intervals. However, in our center, many patients with aSAH underwent only one blood sampling before treatment due to their urgent status. In the future, we intend to conduct prospective studies to further explore the significance of inflammatory markers in aSAH by taking repeated measurements within 24 h of admission. Some inflammatory indicators, such as interleukins and C-reactive protein, were not enrolled in our study. This study lacks a prospective and multicenter design. Future multicenter prospective randomized studies with larger sample sizes should be conducted to address this issue.

## Conclusion

An increased F-NLR score at admission was associated with poorer functional outcomes for patients with aSAH. This biomarker could be considered in multicenter prospective studies for short-term and long-term outcomes.

## Data availability statement

The raw data supporting the conclusions of this article will be made available by the authors, without undue reservation.

## Author contributions

JY contributed to the conception and design of the study, had full access to all the data in the study, and take responsibility for the integrity of the data and the accuracy of the data analysis. YH, HY, and RC contributed to the acquisition of data. HL and YH contributed to the analysis and interpretation of the data. All authors participated in manuscript writing, revision, and approved the submitted version.

## Conflict of interest

The authors declare that the research was conducted in the absence of any commercial or financial relationships that could be construed as a potential conflict of interest.

## Publisher's note

All claims expressed in this article are solely those of the authors and do not necessarily represent those of their affiliated organizations, or those of the publisher, the editors and the reviewers. Any product that may be evaluated in this article, or claim that may be made by its manufacturer, is not guaranteed or endorsed by the publisher.
